# Fungal Keratitis Following Application of Dehydrated Amniotic Membrane (Omnigen) With (OmniLenz) Bandage Contact Lens

**DOI:** 10.1155/crop/3157153

**Published:** 2026-04-28

**Authors:** Bayan Ali Alsaif, Albaraa T. Alfaraidi, Rawan Alshabeeb

**Affiliations:** ^1^ Anterior Segment Division, King Khaled Eye Specialist Hospital, Riyadh, Saudi Arabia, kkesh.med.sa; ^2^ Department of Ophthalmology, College of Medicine, Imam Abdulrahman Bin Faisal University, Dammam, Saudi Arabia, iau.edu.sa; ^3^ Ophthalmology Department, King Fahad Armed Forces Hospital, Jeddah, Saudi Arabia, kfafh.org

**Keywords:** *Candida parapsilosis*, contact lenses, keratitis, keratoplasty, penetrating

## Abstract

**Background:**

Amniotic membrane is a common treatment option for several corneal and conjunctival conditions. There are several methods for preserving amniotic membrane, and each involves some compromise of the tissue integrity. Dehydrated amniotic membrane has been used for several indications, including microbial infections, aniridia, chemical burns, and limbal stem cell deficiency, as in Steven–Johnson syndrome. We report a case of fungal keratitis following the application of a sutureless dehydrated amniotic membrane using specialized bandage contact lens, OmnioLenz (NuVision Biotherapies Ltd., United Kingdom).

**Case Report:**

A 34‐year‐old male with keratoconus who had undergone deep anterior lamellar keratoplasty presented with a recurrent epithelial defect at the graft–host junction that was not responding to conservative management. A sutureless dehydrated amniotic membrane was applied but the patient developed fungal keratitis in the central cornea. The infiltrate culture revealed *Candida parapsilosis*. The patient was started on antifungal and antibacterial topical eyedrops and showed favorable response to topical antimicrobial agents on follow‐up.

**Conclusion:**

High suspicion and close observation are recommended for patients who underwent keratoplasty, especially in the presence of multiple risk factors such as dehydrated amniotic membrane transplant, to detect any signs of infection in a timely manner and thereby prevent vision‐threatening complications.

## 1. Introduction

Amniotic membrane transplantation (AMT) is a common treatment option for several corneal and conjunctival conditions. There are several methods for preserving amniotic membrane; however, each involves some compromise of tissue integrity. Cryopreservation remains the most common technique of preservation. Freeze drying involves cooling the amnion to −80°C, and then a process of sublimation follows to remove the water from the tissue. Dehydrated amniotic membranes are processed by air or heat to remove the moisture for tissue dehydration. Gamma irradiation is used to sterilize the tissue in freeze drying and dehydrated tissue [1]. Dehydrated amniotic membrane has been used for several indications, including the treatment of persistent epithelial defect, microbial infections, aniridia, chemical burn, and limbal stem cell deficiency, as in Steven–Johnson syndrome [2, 3] . We report a case of fungal keratitis following the application of sutureless dehydrated AMT using a specialized OmnioLenz (NuVision Biotherapies Ltd., United Kingdom) bandage contact lens (BCL).

## 2. Case Report

A 34‐year‐old male not known to have any systemic disease underwent deep anterior lamellar keratoplasty (DALK) for keratoconus in January 2022. The postoperative course was unremarkable, and the epithelial defect completely healed on the 7th day after surgery. Three months postoperatively, the patient presented with a 3 × 2‐mm epithelial defect at the graft–host junction, which was managed with contact lens and topical lubricants. The patient had an uncorrected distance visual acuity (UCDVA) of 20/60 and best‐corrected distance visual acuity (BCDVA) 20/40. One week later, the epithelial defect was healed, and the BCL was removed. He was on topical prednisolone acetate 1% twice daily. Five months postoperatively, the patient presented with a loose suture inferotemporal. Corneal resuturing was performed, and a BCL was applied then removed after 3 days without any complication seen. The patient was seen 1 month later with a recurrent loose suture and a corneal epithelial defect. The suture was removed, and an Omnigen (NuVision Biotherapies Ltd., United Kingdom) was mounted on the OmnioLenz BCL. The prednisolone acetate was changed to fluorometholone, a less potent topical steroid, and the patient was started on prophylactic topical moxifloxacin eye drops of four times daily and lubricant eyedrops. The patient presented to the clinic 3 weeks later and reported improvement of symptoms; therefore, the contact lens was removed. Examination showed a resorbed amniotic membrane and a healed epithelial defect in the graft–host junction with quiet conjunctiva along with multiple small central subepithelial corneal opacities that were overlooked during examination with an intact epithelium and no staining as well as a deep quiet anterior chamber. One month later, the patient presented with mild eye redness and decreased vision. On examination, there was a severe conjunctival injection, small central corneal infiltrates, and a mild anterior chamber reaction. Corneal scraping was obtained for (Gram, Giemsa, KOH, Gorcotts′s methenamine silver, and calcofluor‐white) stains, cultures (blood, chocolate, Sabouraud dextrose, and nonnutrient agars), and polymerase chain reaction (PCR) for herpes simplex virus DNA. The impression was microbial keratitis confirmed with cultures and stains that were positive for *Candida parapsilosis* and *Staphylococcus epidermidis* (Figure 1a). PCR was negative for herpes simplex virus. The patient was treated with topical antifungal eyedrops consisting of amphotericin B 0.15% and voriconazole 1%, along with fortified antibiotics eyedrops ceftazidime 50 mg/mL and vancomycin 25 mg/mL. The patient responded to treatment but developed corneal scarring with neovascularization and was maintained on a long‐term regimen of amphotericin B 0.15% and voriconazole 1% twice daily. The patient was started on topical steroid eyedrops 2 months after the diagnosis of fungal keratitis. On follow up, the patient had a UCDVA with pinhole of 20/60 and showed remarkable improvement of symptoms along with resolution of the corneal infiltrate (Figure 1b). The patient′s visual acuity was checked at each visit of follow‐up.

Figure 1(a) A slit lamp photo at initial presentation showing a central infiltrate in the graft that was culture proven *Candida parapsilosis*. (b) A slit lamp photo on last follow‐up showing scarred infiltrate and healed epithelial defect.

**Figure:**
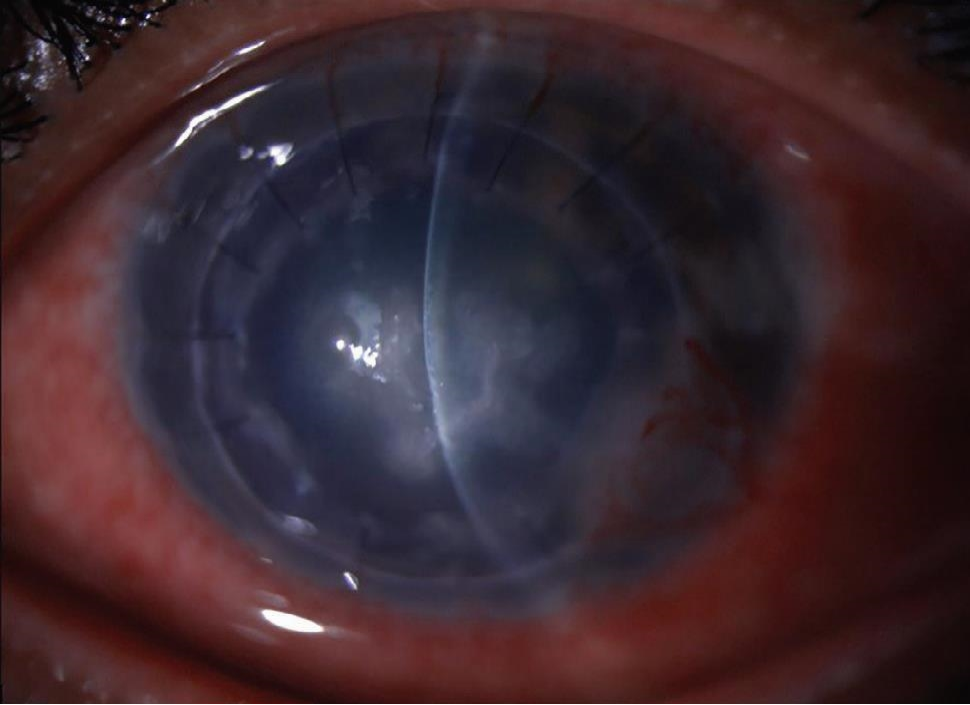
(a)

**Figure:**
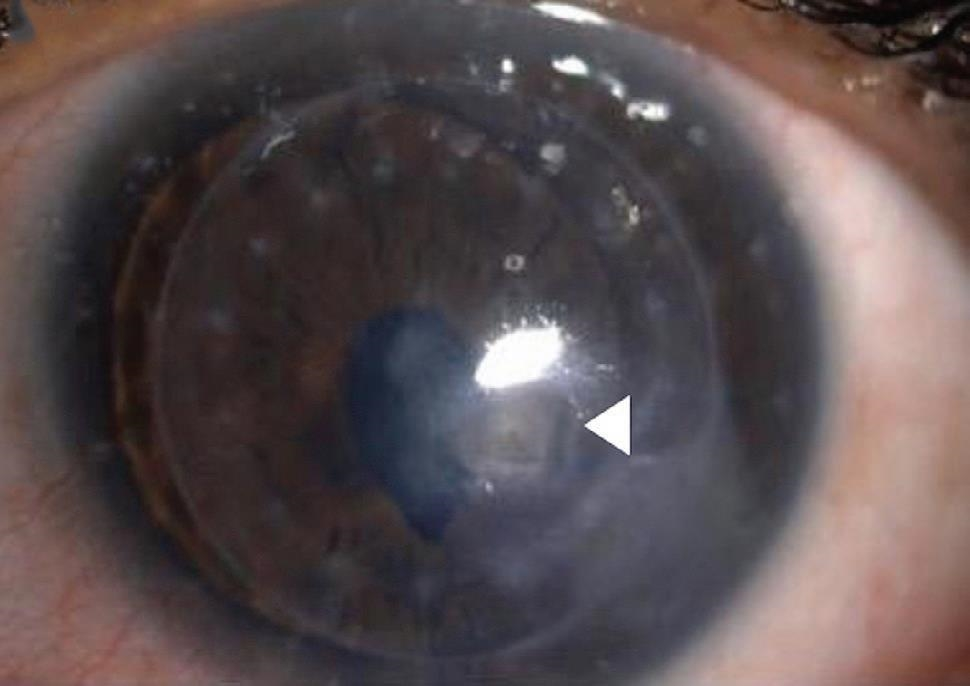
(b)

## 3. Discussion

Multiple commercially available amniotic membranes exist in the market that are effective for office‐based applications in the management of various ocular surface conditions that have demonstrated favorable outcomes [2, 4, 5]. NuVision (Nottingham, United Kingdom) manufactures both Omnigen and OmniLenz utilizing their Tereo processing technique. This technique involves a modified low‐temperature vacuum dehydration process of the amniotic membrane supplemented by lyoprotective saccharides and the antioxidant epigallocatechin. These methods facilitate the storage and distribution of processed amniotic membranes at ambient temperatures while preserving numerous beneficial properties. It can be stored at temperatures ranging from 2°C to 25°C. The preparation of the amniotic membrane tissue by gamma irradiation is believed to help in preventing microbial keratitis after its application, but in the presented case, we reported the possibility of developing an infection despite the preparation process involved in dehydrated AMT.

Microbial keratitis has been reported following the application of different types of amniotic membranes [6, 7]. Case reports have documented instances of recurrent fungal keratitis following AMT, indicating that although recurrence is rare, it remains a recognized complication. Two cases have been reported following the use of air‐dried dehydrated amniotic membrane for a recurrent epithelial defect, which was complicated by fungal microbial keratitis with cultures yielding growth of *Fusarium* in one case and *Sistotrema biggsiae* in another case [8]. In addition, the occurrence of microbial keratitis following the use of cryopreserved amniotic membranes has also been observed [7]. *C. parapsilosis* is one of the most common fungi cultured from the normal human eye [9]. The use of topical steroids, prolonged BCL use, and presence of AMT increased the risk of microbial infection despite having intact epithelium in central cornea; moreover, penetrating keratoplasty is a major risk factor for developing *C. parapsilosis* keratitis [10] . In the presented case, the patient was otherwise immunocompetent, with no evidence of ocular surface disease. The recurrent epithelial defect at the graft–host junction may be attributed to uneven suturing depth as the procedure was performed by a fellow ophthalmologist in training.

Postoperatively, the patient was treated with topical steroids but developed a central microbial keratitis following the placement of dehydrated amniotic membrane despite an intact epithelium. The low virulence of *C. parapsilosis* and the use of topical corticosteroids may have predisposed the patient to fungal proliferation without significant inflammation, which can be overlooked especially when it occurs distant from the suspected infection site. However, potential contamination sources may have been present and contributed to the development of fungal keratitis, possibly occurring during stages such as packaging, storage, or clinical handling of the dehydrated amniotic membrane. The favorable outcomes may be attributed to the addition of antimicrobial agents to the amniotic membrane during preparation, which mitigated the severity of the infection.

## 4. Conclusions

Fungal keratitis following keratoplasty has been previously reported in the literature; however, reported cases of fungal keratitis following AMT have predominantly involved fresh or cryopreserved tissues. In contrast, the present case highlights an uncommon occurrence following the use of dehydrated AMT, which is generally considered to carry a lower risk of microbial contamination due to its processing and sterilization methods. However, a high index of suspicion and close postoperative monitoring in patients undergoing transplantation with dehydrated amniotic membrane is recommended to facilitate early detection of potential infectious complications.

**Nomenclature**
AMT: amniotic membrane transplantation
DALK: deep anterior lamellar keratoplasty
VA: visual acuity
UCDVA: uncorrected distance visual acuity
BCDVA: best‐corrected distance visual acuity
BCL: bandage contact lens
PCR: polymerase chain reaction

## Author Contributions

All authors were involved in the conception and design of the paper and the analysis and interpretation of data. B.A.A., R.A., and A.T.A. acquired and interpreted the data. B.A.A. drafted the paper.

## Funding

No funding was received for this manuscript.

## Disclosure

The authors critically revised the paper and approved the final version of the paper submitted for publication. All authors agree to be accountable for all aspects of the work in ensuring that questions related to the accuracy or integrity of any part of the work are appropriately investigated and resolved.

## Ethics Statement

This study was conducted ethically in accordance with the World Medical Association Declaration of Helsinki. This study protocol was reviewed and approved by the Human Ethics Committee/Institutional Review Board (HEC/IRB) at King Khaled Eye Specialist Hospital (KKESH) in Riyadh, Saudi Arabia. A written informed consent was obtained from the patient for publication of the details of their medical case and any images.

## Conflicts of Interest

The authors declare no conflicts of interest.

## Data Availability

All data generated or analyzed in this study are included in this article. Further enquiries can be directed to the corresponding author.
